# PRELIMINARY FINDINGS OF A MINDFUL WALKING PROGRAM TO PROMOTE HEALTH IN AFRICAN AMERICAN OLDER ADULTS

**DOI:** 10.1093/geroni/igae098.3692

**Published:** 2024-12-31

**Authors:** Jenna Dzwierzynski, Meda Zabielaite, Jongwon Lee, Halle Prine, Chih-Hsiang Yang

**Affiliations:** University of South Carolina, Columbia, South Carolina, United States; University of South Carolina, Columbia, South Carolina, United States; University of South Carolina, Columbia, South Carolina, United States; University of South Carolina, Columbia, South Carolina, United States; University of South Carolina, Columbia, South Carolina, United States

## Abstract

African American adults aged 60 and older are nearly twice as likely to develop Alzheimer’s disease and other dementias compared to non-Hispanic Whites. Regular physical activity, like walking, benefits brain health and may delay cognitive decline. Mindfulness training is cost-effective and can be easily integrated with a walking program. This study explored whether 24 sessions of 30-minute mindful walking could improve cognitive function, sleep, depressive symptoms, and physical functioning in older African Americans compared to a control group by using pre-post assessment comparisons. After randomization, the intervention group incorporated mindfulness techniques during each walking session over 12 weeks. The control group did not receive any intervention. At Week 13, both groups were reassessed. The study included 66 participants (34 intervention, 32 control, 91% female). Descriptive statistics at Week 13 indicated that the intervention group had slightly better sleep (4.74 ±.44 vs 5.09 ±.51), perceived cognition (1.21 ±.29 vs 1.81 ±.31), depression (6.32 ±.53 vs 6.66 ±.59), and timed-up-and-go (TUG) (11.09 ±.38 vs 11.95 ±.50) scores compared to the control group. The intervention group also had a higher mean MoCA score compared to the control group (25.18 ±.46 vs 24.31 ±.53), with a p-value of.080. The sample size was insufficient for formal statistical testing, and further research with a larger sample is needed to evaluate the efficacy of mindful walking interventions. The preliminary results suggest that mindful walking may support healthy aging and reduce health disparities among older African Americans.

